# Relapsing Polychondritis Associated With Thyroid Carcinoma: A First Malagasy Case

**DOI:** 10.1155/crot/1456640

**Published:** 2025-04-11

**Authors:** Oliva Henintsoa Rakotonirainy, Mamonjisoa Olivier Andrianiaina, Lalao Nomenjanahary Rakotonirina, Volatantely Ratovonjanahary, Fahafahantsoa Rabenja Rapelanoro

**Affiliations:** ^1^Department of Rheumatology and Dermatology, Joseph Raseta Befelatanana University Hospital, Antananarivo, Madagascar; ^2^Department of Internal Medicine, Joseph Raseta Befelatanana University Hospital, Antananarivo, Madagascar

**Keywords:** auricular chondritis, cancer, relapsing polychondritis, tracheobronchial chondritis

## Abstract

Relapsing polychondritis (RP) is a rare disease characterized by recurrent systemic inflammation affecting cartilaginous tissues and proteoglycan-rich tissues. The disease may present in several clinical variants, which can delay diagnosis. Corticosteroids are the treatment of choice for RP. In rare cases, RP can be paraneoplastic. The association of RP with thyroid cancer is unusual. We report the first Malagasy case of a 48-year-old man with RP type one diagnosed within less than a year, presenting with recurrent chondritis of the auricular cartilage, tracheobronchial chondritis, and recurrent episcleritis. The disease was associated with papillary thyroid carcinoma.

## 1. Introduction

Relapsing polychondritis (RP), also known as chronic atrophic polychondritis, is a rare systemic inflammatory disease characterized by recurrent inflammation of cartilages and tissues rich in proteoglycans (Orpha code: 728) [1, 2]. Since its initial description, approximately a thousand cases have been reported upto 2018 [3]. The average age at diagnosis ranges from 44 to 51 years. Comorbidities associated with autoimmune, hematological, and oncological conditions have been documented in the literature [4–6]. Links between malignancy and RP have been described [2, 7]. The most widely known relationship is that with hematological disorders, particularly myelodysplastic syndromes [2, 7, 8]; however, although less frequently, relationships with solid neoplasia have also been described [8]. We present the first Malagasy case of RP in 48-year-old man. The disease was associated with papillary thyroid carcinoma.

## 2. Case Presentation

A 48-year-old man was admitted to the Rheumatology Department for investigation of unexplained weight loss. The patient had experienced persistent dry cough, progressive weight loss, evening fever, and intermittent inflammatory joint pain, primarily affecting the knees and ankles, over the past 6 months. Complementary investigations revealed no *Mycobacterium tuberculosis* in the sputum. A chest X-ray showed an apical alveolar opacity in the right lung. The patient was initially treated with antibiotics targeting community-acquired pneumonia, followed by antituberculous treatment due to the apical lesion observed on the X-ray. However, after 2 months of antituberculous therapy, there was no notable improvement. In addition, the patient developed progressive hoarseness, painless bilateral ocular redness, persistent pain, and red swelling of the ear auricles.

The patient had a history of recurrent pneumonias. He was a nonsmoker, with no family history of cancer or autoimmune diseases.

**Figure 1:**
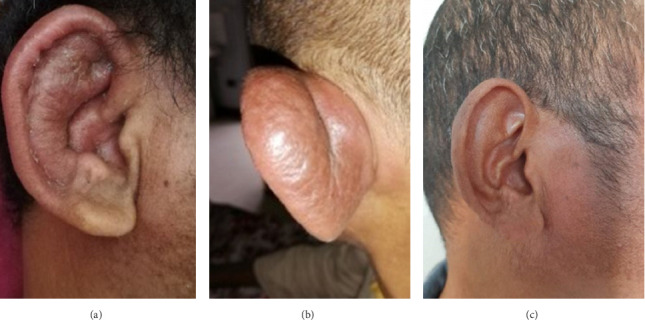
(a-b) Inflammatory swelling of the right ear, sparing the noncartilaginous lobule. (c) Normal appearance of the right ear at 5 months of corticosteroid treatment.

Upon admission, the patient was febrile with a temperature of 38.1°C. Vital signs were stable, with SpO2 at 95% on room air, a respiratory rate of 20 breaths/minute, and blood pressure of 110/60 mmHg. His general condition was impaired, with asthenia (Performance Status two) and weight loss, an BMI of 18 (weight: 46 kg and height: 1.6 m). He exhibited red, painful auricular pavillons, sparing the lobules (Figures 1(a) and 1(b)). There was no hearing loss. His eyes were red, with no exophthalmos, and the ocular redness recurred during hospitalization. He had dysphonia with a hoarse voice, and palpation of the larynx was painful. No nasal deformity was noted. Erythematous, painful subcutaneous nodules, were observed on the limbs. Arthralgia and joint swelling had resolved. No palpable tumor mass was detected.

Biological examinations showed an inflammatory syndrome with CRP at 96 mg/L and an ESR of 165 mm at the first hour. The complete blood count revealed moderate anemia with hemoglobin at 105 g/L, leukocytes at 6 G/L, and platelets at 159 G/L. *Mycobacterium tuberculosis* testing by GeneXpert on sputum was negative. PCR for COVID-19, as well as serologies for HIV, hepatitis B, and hepatitis C, were also negative. Antinuclear antibody (ANA) and ANCA tests were negative. The thyroid function test was normal, with TSH at 1.17 µIU/L, free T3 at 5.2 pmol/L, and free T4 at 15 pmol/L.

**Figure 2:**
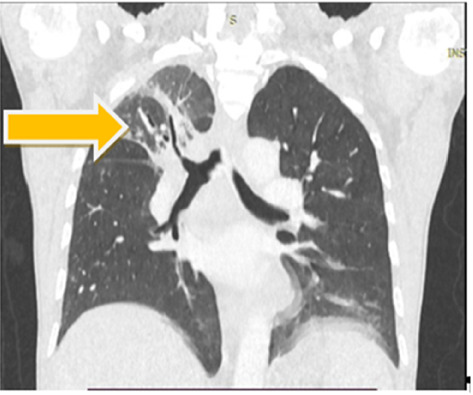
Cervicothoracic CT scan with and without contrast, frontal view showing tracheobronchial wall thickening with right apical atelectasis (yellow arrow).

**Figure 3:**
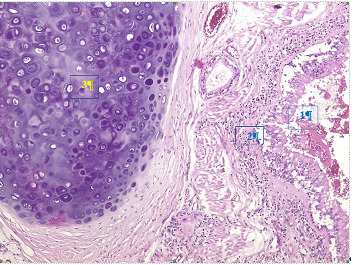
Bronchial biopsy Legends: (1) normal mucosa; (2) chronic inflammation of the stroma; and (3) cartilage Staining: Hematoxylin and eosin Magnification x100. Source: Laboratory of Anatomy and Pathological Cytology of University Hospital Center Jospeh Ravoahangy Andrianavalona in Antananarivo, Madagascar.

Cervicothoracic CT scan, performed with and without contrast injection, revealed tracheobronchial wall thickening with right apical atelectasis (Figure 2) and a thyroid nodule. Bronchoscopy showed congestion and thickening of the right interlobar and segmental ridges. Histopathological examination of the tracheal (Figure 3) and auricular biopsies revealed an infiltrate dominated by lymphocytes, with the presence of neutrophils and monocytes at the chondro-dermal junction. Ophthalmological consultation at each episode of ocular signs revealed bilateral episcleritis.

**Figure 4:**
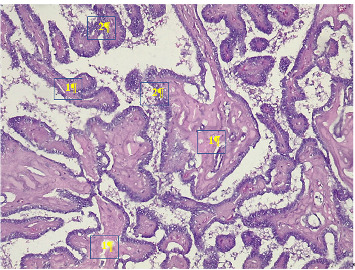
Papillary thyroid carcinoma on surgical specimen Legends: (1) papillary axes and (2) thyrocytes with a frosted glass appearance, overlapping staining: Hematoxylin and eosin Magnification x100. Source: Laboratory of Anatomy and Pathological Cytology of University Hospital Center Jospeh Ravoahangy Andrianavalona in Antananarivo, Madagascar.

Given this clinical presentation, the diagnosis of RP type one was made due to the recurrent auricular chondritis, tracheobronchial chondritis observed on CT scan and bronchoscopy, and recurrent episcleritis. The patient was treated with corticosteroids at a dose of 1 mg/kg/day. The auricular chondritis resolved after 5 months of corticosteroid treatment (Figure 1(c)). However, dysphonia persisted. At 7 months into the treatment and during the tapering phase of corticosteroids, a relapse of bilateral auricular chondritis and red eyes was observed. A bolus of methylprednisolone at 10 mg/kg/day for 3 days was administered. A total thyroidectomy was performed, and the pathological examination of the resected specimen revealed papillary thyroid carcinoma (Figure 4). The patient received radiotherapy and oncological follow-up. The clinical evolution showed complete remission of chondritis. The patient was lost to follow-up. Two years later, the family informed us by phone that there had been no recurrence of chondritis, but the patient had died from acute abdominal pain.

## 3. Discussion

This case represents the first Malagasy observation of RP. The disease was associated with papillary thyroid carcinoma. RP is a rare systemic autoimmune disease, with an estimated annual incidence of 0.7–3.5 per million person-years [9, 10]. Its prevalence is estimated between 4.5 and 20 cases per million adults [11]. It is a systemic disease primarily affecting cartilaginous structures (ears, nose, and tracheobronchial tree) as well as proteoglycan-rich tissues (joints, eyes, vascular walls, skin, and kidneys). The clinical presentations of RP are varied, manifesting as recurrent inflammation of cartilaginous and proteoglycan-rich tissues. Auricular chondritis and polyarthritis are considered hallmark features of the disease but are not pathognomonic. At the onset of the disease, auricular chondritis is absent in nearly half of the cases and polyarthritis is present in only about 33% of the cases [1, 12]. Diagnosis is often delayed, which can worsen the patient's prognosis.

Several authors have proposed diagnostic criteria for RP [13, 14]. Michet et al. defined diagnostic criteria with major criteria (nasal chondritis, auricular chondritis, and laryngo-tracheal chondritis) and minor criteria (ocular inflammation, hypoacusis, vestibular syndrome, and nonerosive polyarthralgias). The diagnosis of RP is made when there are two major criteria or one major criterion and two minor criteria [13]. The clinical presentation of RP in our patient was comprehensive, allowing for a prompt diagnosis.

To ensure greater consistency and identify prognostic factors, other classifications of RP have been proposed [15, 16]. Ferrada et al. suggested a classification into three subgroups [15]: Type one is characterized by extensive cartilage lesions, with involvement of the ear and tracheobronchial tree always present; Type two is marked by significant involvement of the lower respiratory tract with less involvement of cranial cartilaginous structures; and Type three is defined by minimal cartilage lesions, including subglottic stenosis and saddle nose deformity, without signs of tracheomalacia or bronchomalacia. RP Type one, like that of our patient, accounts for about 14% of the patients and is characterized by a relatively short diagnostic delay, around 1 year. Dion et al. also proposed three phenotypes of the disease [16]. This classification helps identify mortality factors associated with RP. The first phenotype corresponds to an older patient at diagnosis with a myelodysplastic syndrome, with a mortality rate of 58%. The second phenotype involves younger subjects with involvement of the tracheobronchial or laryngeal tree, with an intermediate survival rate of about 13%, requiring immunosuppressive or biological treatment. The third phenotype, the most common, is characterized by minor symptoms, no myelodysplastic syndrome, and minimal respiratory involvement (3%), with a similar prognosis to phenotypes two and three in terms of survival. Our patient corresponded to the second phenotype. Significant involvement of the tracheobronchial tree is responsible for recurrent pneumonias and repeated stays in the intensive care unit for respiratory distress [17, 18]. In our case, the absence of respiratory distress was likely due to the patency of the tracheobronchial orifices observed during bronchoscopy and the rapid introduction of corticosteroid treatment. The dysphonia may be related to chondritis of the laryngeal cartilages [17].

RP can be associated with various comorbidities, including autoimmune diseases and cancers, such as myelodysplastic syndrome, lymphoma, and carcinomas [8, 19]. Ludvigsson et al. reported that RP is associated with cancer in 25% of the cases [4]. Solid cancers associated with RP can affect different organs (lungs, colon, skin, breast, prostate, soft tissue sarcomas, and pituitary gland) [6, 8]. RP associated with thyroid cancer is rare, with thyroid conditions associated with RP primarily being autoimmune diseases such as Graves' disease or Hashimoto's thyroiditis [5]. The absence of recurrence of polychondritis after the specific management of thyroid cancer leads us to conclude a paraneoplastic polychondritis.

Currently, there is no consensus treatment for RP. The choice of treatment depends on the clinical manifestations, severity of RP, type of organic damage, and comorbidities [8, 11]. Low-dose corticosteroids are generally accepted as first-line treatment [3, 11, 20]. The route of administration (systemic, bolus, and local) depends on the clinical manifestations. Minor forms can be treated with nonsteroidal anti-inflammatory drugs or colchicine as a maintenance treatment. Corticosteroid-dependent, corticosteroid-resistant, or severe forms require treatment with immunosuppressants or biotherapy [8, 20].

## 4. Conclusions

RP is a rare systemic inflammatory disease primarily affecting cartilage. The presence of characteristic symptoms involving cartilaginous and proteoglycan-rich tissues (such as auricular chondritis, polyarthritis, and recurrent pneumonia) facilitates the diagnosis of RP. In rare instances, RP can present as a paraneoplastic syndrome associated with carcinoma. This case report contributes to the existing knowledge on this condition.

## Data Availability

The data that support the findings of this study are available from the corresponding author upon reasonable request.
